# Elevated Ambient Temperature Associated With Reduced Infectious Disease Test Positivity Rates: Retrospective Observational Analysis of Statewide COVID-19 Testing and Weather Across California Counties

**DOI:** 10.2196/57495

**Published:** 2024-12-12

**Authors:** Nicholas Wing-Ping Kwok, Joshua Pevnick, Keith Feldman

**Affiliations:** 1 Department of Medicine, Cedars-Sinai Medical Center, Los Angeles, CA, United States; 2 Department of Biomedical Sciences, Cedars-Sinai Medical Center, Los Angeles, CA, United States; 3 Department of Pediatrics, Children's Mercy Kansas City, 2401 Gillham Road, Kansas City, MO, 64108, United States, 1 816-302-0512; 4 Department of Pediatrics, University of Missouri–Kansas City School of Medicine, Kansas City, MO, United States

**Keywords:** body temperature, BT, fever, febrile, feverish, ambient temperature, environmental factor, environmental context, environmental, environment, COVID-19, SARS-CoV-2, coronavirus, respiratory, infectious, pulmonary, COVID-19 pandemic, pandemic, diagnostics, diagnostic test, diagnostic testing, public health surveillance

## Abstract

**Background:**

From medication usage to the time of day, a number of external factors are known to alter human body temperature (BT), even in the absence of underlying pathology. In select cases, clinical guidance already suggests the consideration of clinical and demographic factors when interpreting BT, such as a decreased threshold for fever as age increases. Recent work has indicated factors impacting BT extend to environmental conditions including ambient temperature. However, the effect sizes of these relationships are often small, and it remains unclear if such relationships result in a meaningful impact on real-world health care practices.

**Objective:**

Temperature remains a common element in public health screening efforts. Leveraging the unique testing and reporting infrastructure developed around the COVID-19 pandemic, this paper uses a unique resource of daily-level statewide testing data to assess the relationship between ambient temperatures and positivity rates. As fever was a primary symptom that triggered diagnostic testing for COVID-19, this work hypothesizes that environmentally mediated BT increases would not reflect pathology, leading to decreased COVID-19 test positivity rates as temperature rises.

**Methods:**

Statewide COVID-19 polymerase chain reaction testing data curated by the California Department of Public Health were used to obtain the daily number of total tests and positivity rates for all counties across the state. These data were combined with ambient temperature data provided by the National Centers for Environmental Information for a period of 133 days between widespread testing availability and vaccine approval. A mixed-effects beta-regression model was used to estimate daily COVID-19 test positivity rate as a function of ambient temperature, population, and estimates of COVID prevalence, with nested random effects for a day of the week within unique counties across the state.

**Results:**

Considering over 19 million tests performed over 4 months and across 45 distinct counties, adjusted model results highlighted a significant negative association between daily ambient temperature and testing positivity rate (*P*<.001). Results of the model are strengthened as, using the same testing data, this relationship was not present in a sensitivity analysis using random daily temperatures drawn from the range of observed values (*P*=.52).

**Conclusions:**

These results support the underlying hypothesis and demonstrate the relationship between environmental factors and BT can impact an essential public health activity. As health care continues to operate using thresholds of BT as anchor points (ie, ≥100.4 as fever) it is increasingly important to develop approaches to integrate the array of factors known to influence BT measurement. Moreover, as weather data are not often readily available in the same systems as patient data, these findings present a compelling case for future research into when and how environmental context can best be used to improve the interpretation of patient data.

## Introduction

Body temperature (BT), together with pulse rate, blood pressure, and respiratory rate, constitutes one of the four fundamental “vital signs” essential for assessing and monitoring an individual’s health [1]. Given established relationships between abnormal BT and many pathologic conditions, BT is obtained at nearly every health care encounter. Moreover, with the limited equipment required for the collection, BT has become a key element in public health surveillance, particularly in the case of infectious diseases (eg, influenza).

Current clinical practice defines normal adult BT between 36.16 and 37.02 °C (97.09 and 98.64 °F [2]), commonly anchoring abnormality at 2 thresholds: hypothermia (low BT, ≤36 °C) [3] and fever (elevated BT, ≥38 °C [4]). These values are endorsed by the Centers for Disease Control and Prevention (CDC) and are often used as screening criteria, part of differential diagnoses, or in evidence-based practice pathways [5-7]. Unfortunately, the contextualization of measured BT against these thresholds represents a nontrivial comparison.

Ranging from age to medication use, to acute and chronic diagnoses, it is now well recognized that BT varies in response to an array of clinical and demographic factors [8,9]. Clinicians, in turn, have begun to consider such information when interpreting an individual’s BT. For example, developing practice guidelines that account for individuals’ decreased ability to mount fever with increased age [10]. To date, such efforts have largely centered on intrinsic elements of an individual and their health readily available during an encounter. Yet an emerging body of work has prompted questions about the need to consider extrinsic, environmental factors, specifically ambient temperature.

While commonly associated with conditions such as heat stroke [11] or hyperthermia [12], foundational work by Obermeyer et al [8] demonstrated that changes in ambient temperature represent an independent factor linked to changes in measured BT in healthy adults. This effect has been amplified as age increases [13], potentially tied to the effects of impaired thermoregulation. Moreover, the confounding nature of ambient temperature on BT interpretation is compounded by the frequent use of noninvasive temperature measurement techniques (oral and tympanic), which have also been shown to be highly influenced by ambient temperature [14].

Nonetheless, the effect sizes between associations of BT and ambient temperature are small to moderate, and it remains unclear if their consideration would have a meaningful impact on real-world health care practices. The work presented in this paper addresses this exact question, leveraging the unique testing and epidemiologic data sharing efforts associated with the SARS-CoV-2 (COVID-19) pandemic. Given early studies widely identified fever as a primary symptom (>83.5% of cases) associated with COVID-19 infection [15], elevated BT became a common driver for individuals to seek medical testing. Using publicly available testing and weather data, this study measures the association between daily maximum ambient temperature and COVID-19 test positivity rate across 58 California counties over a period of 133 days from the time public COVID-19 testing was widely available until initial vaccine use began.

We hypothesized higher ambient temperatures (and expected associated increases in individual BT) would lead to increased suspicion of febrile illness. However, as the shift in BT would be environmentally driven, and not pathologic, this increase would manifest in a negative relationship between ambient temperature and test positivity rate. In doing so, we illustrate a clear example of the impact of BT-altering factors on a common real-world public health activity and present a discussion of how such information can be integrated into BT interpretation workflows.

## Methods

### Study Design and Population

This work presents an observational retrospective analysis of daily COVID-19 testing data across the state of California. As a direct result of efforts to monitor disease spread and community risk, COVID-19 testing data were covered under “diseases of public health importance” and were collected at the state level from all laboratories under the California Code of Regulations. This highly granular and longitudinal testing effort throughout the pandemic, coupled with the understanding that febrile illness represented a clinical indication for COVID-19 illness, provided a unique and well-suited case for examining the relationship between ambient temperature and testing.

This work evaluated testing conducted between August 1, 2020, and December 11, 2020, representing a study period of 133 days. This nearly 6-month window is able to capture seasonal variability, spanning a wide range of average monthly temperatures across the state (26.1 °C in August 2020 to 7.5 °C in December 2020) and allowing for analysis across a comprehensive distribution of temperatures. Testing data represented gold-standard PCR (polymerase chain reaction) and were reported by the performing laboratories, allowing for aggregation across tests performed in emergency rooms, hospitals, clinics, pharmacies, and other testing centers. The study period itself was selected to help minimize bias from the wide array of socioeconomic and logistic factors inherent to the COVID-19 pandemic.

The start date was chosen to approximate the time when COVID-19 tests were available in all California counties and were no longer being rationed. The end date selection was multifactorial. First, based on the first FDA (US Food and Drug Administration) emergency use authorization (EUA) for a COVID vaccine (EUA for a COVID vaccine was granted to Pfizer on December 11, 2020, and to Moderna on December 18, 2020), this timepoint conservatively approximates the end of the pre-vaccine period. Given that the vaccine status of an individual may impact testing practices and the ability to mount fever, this approach helps to mitigate bias. Additionally, the first COVID-19 variant (Alpha, B.1.1.7) was initially detected in the United States on December 29, 2020. Restricting the analysis to the initial pandemic wave can also help to assure a stable study cohort on which to perform the analysis in this work.

### Data Collection

#### Overview

Data were drawn from 2 primary sources. First, weather data were collected from the United States National Oceanic and Atmospheric Administration (NOAA). Second, daily-level COVID-19 testing data were provided by the state of California. Details surrounding the collection, cleaning, and processing of each data source can be found in the respective subsections to follow.

#### COVID-19 Data

Daily COVID-19 testing data were obtained at the county level from the California COVID-19 State Dashboard [16]. These data were curated by the California Department of Public Health (CDPH) and included all testing data as available across the state (Title 17 of the California Code of Regulations listed COVID-19 as a reportable disease [17]), reported by laboratories through the California Reportable Disease Information Exchange. For each of the 58 counties across the state of California, data included the date specimens were collected, the number of COVID-19 tests submitted, and the number of positive tests. Of note, the CDPH aligned testing data to the specimen collection date, rather than the date results became available, which provided a robust mechanism to assure that the positivity rate is correctly linked to the temperature of a given day. Tests that may have been processed for labs outside of California (location listed as “out of state”) or without a clear indication for the test location (location listed as “unknown” or associated generally to the state, that is, “California”) were excluded.

County population data were sourced from the 2020 California Department of Finances population estimates. The county populations, together with the daily positive test counts, were used to derive an estimate of daily COVID-19 incidence. For a given day, this value is approximated as a sum of daily positive tests over the prior 10 days, divided by the county population. This window was selected to align with CDC recommendations, assuming conservatively that testing occurred on the day of symptom onset [18].

Data were collected for all counties in California. However, to minimize bias in positivity rates resulting from small counties, or irregular testing patterns, a subset of data were excluded as follows. First, counties with a population of fewer than 25,000 were excluded due to multiple days with low rates of testing and low COVID-19 prevalence rates. Second, the distribution of total daily tests was calculated during the study period, and the bottom quartile of days was removed from the data set. This helps to mitigate large effects on positivity due to small absolute changes in positive tests relative to total tests and to exclude situations in which test rationing may have occurred thereby biasing the relationship of testing to positivity rate. Finally, counties that provided testing data for less than half of the total days in the study window were omitted to allow for a more precise estimation of within-county variance for the expected regional clustering in the relationship between positivity and testing.

#### Weather Data

Weather data was collected from 595 NOAA weather stations across the state of California using the National Centers for Environmental Information Climate Data Online tool [19]. For each station, data included a daily timestamp and associated maximum ambient temperature. Also collected were a set of logistical data including each station’s precise geospatial location (latitude and longitude). NOAA temperature data is highly precise and reliable [20]. Stations are equipped with 3 thermometers to compute independent 5-minute averages from 2-second readings from each thermometer. These measurements are then used to derive an hourly temperature and in turn a daily maximum. Consistency checks between sensors are performed as part of data capture, and for each daily measurement quality indicators are provided to indicate potential reliability issues or are directly recorded as “null” if a value could not be computed. Any daily measurements marked as unreliable were removed, and weather stations with no data were excluded from the study.

To associate a daily maximum temperature to county-level testing data, a weighted mean of temperatures from all weather stations within each county was calculated. As counties can span larger geographic areas, weights were created to represent the population size of the ZCTAs (zip code tabulation areas) in which each station resides. To do so, reverse geocoding for station latitudes and longitudes was performed to obtain a zip code for each station using the Nominatim API [21]. Zip code to ZCTA mappings were then retrieved from the 2021 Uniform Data System table, and ZCTA to population mappings were retrieved from the American Community Survey 5-year data [22]. Notably, although each county was composed of multiple weather stations, a percentage of the total population does not live within a ZCTA containing a weather station. To account for the magnitude of these unassigned individuals in the weighting, the remainder of the population was assumed to be evenly distributed throughout the county, with equal exposure to the weather at each station, as shown in Equation 1. Equation 1 demonstrates that the daily maximum ambient temperature for a county is calculated as the sum of the weighted sum of the weather station maximum ambient temperatures (*T*_*i*_) by a fraction of the station population (pop_*i*_) relative to the county population (pop_c_) and the product of the average station temperature with the fraction of a county’s population not associated with a weather station relative to county population. pop_w_ represents the total population associated with a weather station.


(1) $$\begin{array}{l} temp_{amb}=(1/pop_{c})*\sum_{i=1}^{n}\left(T_{i}*pop_{i}\right)+1/(n*pop_{c})*\;\sum_{i=1}^{n}T_{i}*(pop_{c}-pop_{w}) \end{array}$$

### Statistical Analysis

The unadjusted association between temperature and positivity rate was characterized using the Spearman rank correlation coefficient for multiple representative counties (the counties with minimum, median, and maximum populations). To robustly estimate the association between temperature and positivity rate (calculated as the ratio between positive tests and total tests on a given day), a multivariate mixed-effects beta regression was used. Daily positivity rate was estimated with a logit link function, as a function of daily maximum ambient temperature, derived COVID-19 prevalence, and county population. To account for variability in testing practices and community behavior early in the pandemic, random effects were added for the county. Additionally, a random effect for day of the week (DOW) was nested within the county to account for regional testing and potentially different reporting practices (eg, weekend test results were sometimes all reported on Mondays). To aid in model convergence, the prevalence was scaled by a factor of 1000, and the county population was log-transformed to account for the heavy right tail of a few large counties. Positivity rate was adjusted using a scalar transform to avoid zero values [23].

To assess model stability, several diagnostics and post hoc analyses were performed. First, variance inflation factors were computed to assess collinearity between the study variables. Next, a series of model diagnostics were calculated including residual versus predictor plots, a quantile-quantile plot of residuals, an outlier test plot, and a nonparametric dispersion test plot using the standard deviation of fitted and simulated residuals. Finally, as a sensitivity analysis for associations to the max-temperature data, the beta regression was refitted, this time using a randomly drawn daily max temperature between –6.7 and 60 °C, similar to the range of observed temperatures. All other data were left unmodified. All data processing and analysis were performed using R (version 2023.12.0), together with TidyVerse [24], and the GLMM TMB packages [25]. Diagnostics used the DHARMa package [26].

### Ethical Considerations

This study was deemed non-human subjects research. All data were retrieved from public use datasets, and secondary analyses were performed. Therefore, this study did not require institutional review board review per Federal Regulations for the Protection of Human Research Subjects (45CFR 46) [27].

## Results

COVID-19 testing data were reported by the state for each of the 58 total counties. Of those, 1 (Sutter County) did not report temperature data at any weather station during this time period, and 1 county (Del Norte) did not have any zip code associated with its weather stations; both were omitted. Nine counties had populations below 25,000 (Alpine, Colusa, Inyo, Mariposa, Modoc, Mono, Plumas, Sierra, and Trinity) and were omitted. After filtering out days in the bottom 25% of total tests administered (n=123), and for missing temperature values, 4 counties (Calaveras, Glenn, Lake, and Siskiyou) were found to provide data for less than 50% of total days in the study window (66 of 133 days, August 1 to December 11, 2020), and were thus omitted. This resulted in a final cohort of 43 unique counties. An overview of the testing and weather data for these counties can be found in Table 1.

**Table 1.** Overview of county data during the study period.

Data set and characteristic		Value	
**County descriptions**			
	Unique counties, n	43	
	Population (1000s), mean (SD); range	988 (1710); 30‐10,300	
**COVID-19 testing data, mean (SD); range**			
	Mean testing days	122 (15.1); 78‐133	
	Daily total tests	3790 (9420); 123‐128,000	
	Daily COVID-19 positivity rate	0.058 (0.043); 0.000067‐0.36	
	Daily COVID-19 prevalence (per 1000 population)	2.1 (2.6); 0.057‐37	
**NOAA^a^ weather data, mean (SD); range**			
	Number of weather stations	11 (8.7); 2‐42	
	Mean daily maximum ambient temperature (°C)	26.6 (7.3); 2-47.8	

a NOAA: United States National Oceanic and Atmospheric Administration.

**Figure 1.**
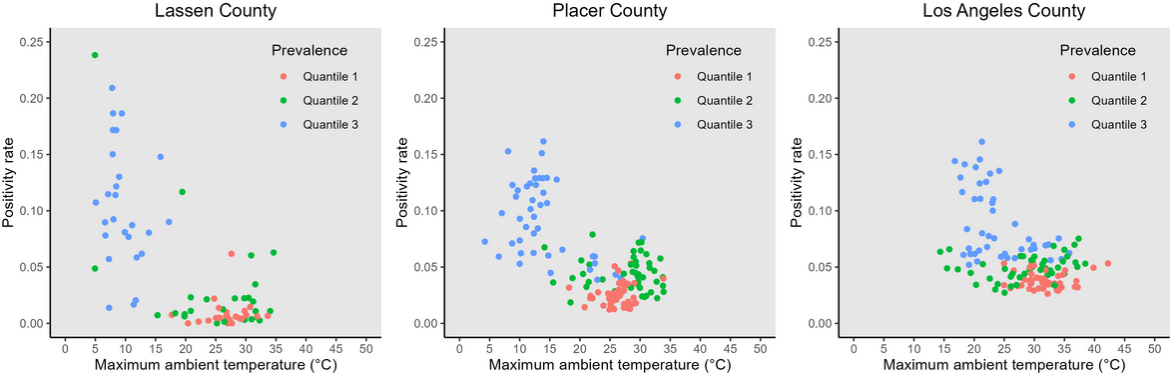
Unadjusted daily COVID-19 positivity rate plotted against daily maximum ambient temperature for the counties with the minimum (Lassen), median (Placer), and maximum (Los Angeles) populations.

For context to the underlying hypothesis, unadjusted plots for daily maximum ambient temperature versus daily COVID-19 positivity rate can be found for the minimum, median, and maximum counties by population (Lassen, Placer, and Los Angeles, respectively) in Figure 1. In all 3 counties, Spearman rank correlation coefficients were negative with *P* values below .01, as shown in Table 2, demonstrating an overall negative correlation between positivity rate and maximum ambient temperature. Adjusted results from the beta regression and primary analysis can be found in Table 3.

**Table 2.** Unadjusted Spearman correlation between daily maximum ambient temperature and daily COVID-19 testing positivity rate of the minimum, median, and maximum counties by population during the study period.

County	Spearman rank correlation (ρ)	*P* value
Lassen	−0.61	<.001
Placer	−0.49	<.001
Los Angeles	−0.38	<.001

**Table 3.** Adjusted coefficients and 95% CIs of beta regression estimating daily COVID-19 positivity rate. COVID-19 prevalence is represented as #/1000, and the county population is log-transformed.

	Odds ratio (95% CI)	*P* value
Intercept^a^	0.0059 (0.0012-0.029)	<.001
Daily maximum ambient temperature^a^	0.993 (0.992-0.994)	<.001
Daily COVID-19 prevalence	1.14 (1.13-1.15)	<.001
County population	1.2 (1.1-1.4)	<.001

a Coefficients reflect analysis of temperature in °F, as reported in the underlying data source.

Even after adjustment for COVID-19 prevalence and county population, the association of daily maximum ambient temperature was found to be significant (α<.05), presenting with an odds ratio lower than 1 (0.993), implying an inverse relationship to testing positivity rate. Across the state, there was significant variability in the average COVID-19 positivity rate between counties, and to a lesser extent to the DOW on which testing occurred, as noted by the variance of the random effects, 0.28 and 0.025, respectively.

Overall, the model was found to be stable. Variance inflation factors were found to be low (1.36, 1.36, and 1 for temperature, prevalence, and logarithm of the population, respectively) suggesting minimal collinearity. Further, Pearson residuals were calculated and found to be below 3. Complete model diagnostics can be found in Multimedia Appendix 1. Finally, when the model was replicated using random maximum daily temperatures, the association between temperature and positivity rate was no longer significant, with a coefficient of 0.00031 (95% CI −0.00064 to 0.0013; *P*=.52). This in turn provides evidence that the results highlighted here present associations that are products of observed data, and not simply an artifact of the studies large sample size.

## Discussion

### Principal Results

Using more than 19 million COVID-19 PCR tests collected over a period of 5 months, this study identified an inverse relationship between daily maximum ambient temperature and COVID-19 testing positivity rate. Replication of this analysis using identical testing data, but randomized temperatures, does not produce this relationship, offering supporting evidence to the hypothesis that the effects of ambient temperature on BT may not be widely considered in the context of seeking public health infectious disease testing.

Prior literature has established that measured BT is associated with ambient temperature levels, even in individuals not suspected of having pathologic BT derangements [8]. At a fundamental level, the effects of ambient temperature on the human body are known to be driven by several mechanisms that include both thermodynamic principles of metabolic heat production and physiologic consequences of thermoeffector response [28]. Beyond the effects of thermoregulation on core BT, ambient temperatures can influence BT through bias introduced into the measurement itself. Ambient temperature has been notably shown to impact noninvasive measurement tools such as infrared thermometers [29,30]. The bias of ambient temperature on BT measurement has been found to be rapid, occurring within as little as 20 minutes of exposure to changes in ambient temperature, leading to a significant degree of misalignment from true BT reaching over 1 degree change over this time period [31,32].

However, BT is not used in isolation, and downstream implications on health care practice arising from this association between ambient temperature and BT have not been well explored. In the context of decision points for testing and screening, the results of this study indicate that such a relationship may exist. However, we recognize this study is not designed to elucidate the mechanism by which it impacts this practice. Based on findings in the primary and sensitivity analyses, we posit that higher ambient temperatures resulted in elevated BTs (either directly to core temperature due to exposure or through biased measurement), which led individuals to seek out or be referred for testing that reduced positivity rates on warmer days.

Findings also highlighted a positive association between positivity rate and county population, suggesting that even when holding prevalence and temperature constant, testing patterns may differ in larger counties, where perhaps individuals may be more likely to seek testing due to an understanding of their population density and exposure risk. As we move beyond quantifying the deterministic relationships governing BT and ambient temperature, this represents an important premise that studies involving public health practices must also consider behavioral and policy-related factors. With respect to the testing outcomes explored in this work, the positivity rate may be influenced by guidance on who should seek testing and when, as well as structural elements such as the availability or ease of access of tests to a specific population.

Advantageously, the extraordinary circumstances of the COVID-19 pandemic allow us to capture such details at a level not historically available, strengthening confidence in the study procedures. California was a leader in public health testing efforts. Under CDPH guidance (NR20-160) starting in July 2020, prioritization of testing was expanded not only to individuals in health care settings (tier 1) but also to any individual with signs and symptoms (tier 2, which in this case would include elevated BT). By setting the study window to begin in August 2020, we captured all PCR testing statewide under updated reporting guidelines and took further steps to isolate a period of time during which testing was readably available to assure tests were available (removing the lowest quartile of testing days and removing counties with small populations) to minimize effects of rationing.

The presence of associations does not provide insight into their use in practice. The ability to capture and aggregate necessary ambient temperature at the point-of-care remains an open technical and workflow challenge. Nonetheless, efforts are emerging to develop algorithmic corrections for BT due to external perturbations, including for ambient temperature [33]. From a workflow perspective, it is worth noting that bias arising from ambient temperature is not unique to BT. Fluctuations in ambient temperature have also been linked to changes in clinical measurements such as blood pressure [34]. It has also been shown to impact a number of common laboratory tests and key cardiovascular biomarkers [35,36]. Continuing to build on insights around how ambient temperature impacts other health measurements may provide a foundation for best practices in the interpretation and use of BT.

### Strengths and Limitations

Strengths of this study include the large sample size and use of labs for which reporting was mandated statewide by a public health department. Mandatory reporting of lab data by the CDPH during the study period aids in minimizing bias in reporting by site, and the usage of robust PCR testing provides high-quality testing results. Weather data were also highly reliable using hourly data reported from multisensor NOAA stations with robust protocols for data quality.

Despite these strengths, the nature of this study presents several limitations, especially at the patient level. First, we cannot capture other individualized BT-altering factors (eg, demographics or comorbidities), and stratification by clinical presentation was not possible. We were also unable to account for other environmental factors such as wind chill for more precise temperature estimates, as only a small subset of stations provided such data. At a testing level, although all testing was performed using PCR, collection type was not available (ie, nasopharyngeal, nasal, and saliva). While PCR remains the gold standard in COVID-19 testing, recent work has shown differences in sensitivity based on site [37], which may have impacted positivity rates. Additionally, we did not have access to the modality of BT measurement (eg, oral vs forehead) which may have been differentially influenced by ambient temperature. Finally at a policy level, although we have taken steps to provide as clean an analysis time period as possible, accounting for county-level variance with random effects models, individual policy details (mask mandates, social gathering criteria, etc) are not reported or available at day-to-day level modeled in this work. We remain confident in the findings, however, as this work is designed to capture average effects across an immense population over a nearly 3-month period and includes a wide array of testing locations spanning various illness acuity levels. Finally, in our estimation of prevalence, the 10-day window was based on CDC recommendations for masking. Insight into living arrangements and clinical severity (immunocompromised patients or those with severe COVID) may have resulted in better accounting for differences in viral transmission and the ability to contribute to future incident cases and future prevalence.

### Conclusions

Our finding of an inverse relationship between maximum ambient temperature and COVID-19 test positivity rate suggests that higher maximum ambient temperature may have caused higher BT, resulting in increased testing for infection. Even though ambient temperature is known to affect BT, current efforts to consider ambient temperature in BT interpretation are nearly nonexistent. As such, we suspect that lack of consideration of ambient temperature caused this lower test positivity rate on hotter days. Future research should endeavor to investigate this association more throughly, including the study of its mechanism. Learning more about the effect of ambient temperature on BT and other tests in health care will enable improved consideration of these relevant factors and thereby allow for optimized interpretation of test results.

## Supplementary material

10.2196/57495 Multimedia Appendix 1 Analytical diagnostics.

## Abbreviations:

BT: body temperature
CDC: Centers for Disease Control and Prevention
CDPH: California Department of Public Health
DOW: day of the week
EUA: emergency use authorization
FDA: US Food and Drug Administration
NOAA: United States National Oceanic and Atmospheric Administration
PCR: polymerase chain reaction
ZCTA: zip code tabulation area
