# Supplementary material for: Elevated Ambient Temperature Associated With Reduced Infectious Disease Test Positivity Rates: Retrospective Observational Analysis of Statewide COVID-19 Testing and Weather Across California Counties
Source: JMIR Public Health Surveill. 2024 Dec 12;10:e57495. doi: 10.2196/57495 (PMC11656991; doi:10.2196/57495)
Supplement: Multimedia Appendix 1 [file publichealth-v10-e57495-s001.pdf]

Supplementary Information

Table of Contents

County-Level Unadjusted Association of COVID-19 Test Positivity Rate and Maximum Ambient Temperature .. 2
Modeling COVID-19 Test Positivity Rate Using Multivariate Beta Regression 2
Parameter Transformations *4*
Final Model *5*
Model Diagnostics *6*
Variation Inflation Factors (VIF) 6
Q-Q Plot 7
Outlier Test 8
Dispersion Plot 9
Residual vs Predictor Plots 10
References 13

County-Level Unadjusted Association of COVID-19 Test Positivity Rate and Maximum Ambient Temperature

To assess for a linear association between unadjusted COVID-19 test positivity rate against maximum ambient temperature for the counties with the minimum, median, and maximum populations, Spearman's rank correlation coefficient was calculated using a two-sided null hypothesis (i.e. null hypothesis is that the Spearman's coefficient is zero). Computationally the calculation was performed using cor.test function in the R stats package version 4.1.3. [1]

The Spearman rank correlation coefficient correlates the ranks between two variables in a nonparametric fashion, and can take values between –1 and +1. Since tied ranks were known to be present, the Spearman coefficient was calculated by converting both variables to the ordinal scale, and then applying the formula for product moment correlation to the ranked data. [2] The product moment correlation formula is shown in Equation 1, in which r_{xy} is the Spearman rank correlation coefficient, X is the maximum ambient temperature, Y is the COVID-19 test positivity rate, s_x is the standard deviation of the maximum ambient temperature, s_y is the standard deviation of the COVID-19 test positivity rate, and n is the number of ranks. [3]

$$\boldsymbol{r_{xy}} = \frac{\sum_{i=1}^{n}(\boldsymbol{X_i} - \boldsymbol{X_{avg}})(\boldsymbol{Y_i} - \boldsymbol{Y_{avg}})}{\boldsymbol{s_x s_y}(\boldsymbol{n} - \mathbf{1})} \quad (1)$$

Due to the presence of tied ranks, calculation of an exact p-value was not possible. An inequality for the p-value is given in the table for each county.

Modeling COVID-19 Test Positivity Rate Using Multivariate Beta Regression

Multivariate beta regression models the response of continuous variates with values in the standard unit interval. This response of the variates is assumed to be beta-distributed. Major advantages of this technique include being able to interpret the regression parameters in terms of the mean of the response variable, allowing for heteroskedastic models and asymmetry, and the flexibility of shapes allowed by the beta density, including significant skew [4]. Since the daily COVID-19 test positivity rate is a proportion between daily positive tests and daily total tests in a given county and has the potential for skew toward low or high positivity rates, it is well suited to regression analysis with multivariate beta regression. The related probability distribution function is shown in Equation 2, in

which $G(.)$ is the gamma function, $\mu$is the mean of y, and $\alpha$is the precision parameter. Notably 0 < y < 1, 0 < μ < 1, and α > 0. The precision parameter is also held constant for all observations.

The expected value and variance equations are shown in Equation 3 and 4 respectively.

$$F(y) = y^{\mu\alpha-1} \frac{(1-y)^{(1-\mu)\alpha-1} G(\alpha)}{G(\mu\alpha) * G(\alpha(1-\mu))} \tag{2}$$

$$E(y) = \mu \tag{3}$$

$$VAR(y) = \frac{\mu\,(1-\mu)}{1+\alpha} \tag{4}$$

The logit link function was chosen to link the mean to the regression parameters. Logit link functions are generally preferred when the dependent variable is bounded between 0 and 1 (as in this case with the COVID test positivity rate), and has the additional advantage of allowing interpretation of the parameters as log odds ratios. The beta regression model and logit link function is shown in equation 5 and 6 respectively. B represents the unknown regression parameters in vector form, x_i represents the regressors (or independent variables) in vector form.

$$g(\mu_i) = x_i{}^T * B \tag{5}$$

$$Logit\ g(\mu) = \log\left(\frac{\mu}{1-\mu}\right) \tag{6}$$

Parameter estimation was performed by maximum likelihood. For a formal mathematical definition of the beta regression model, please see [4]. The R package glmmTMB (version 1.1.5) was used to implement the beta regression model, which uses the PORT routine optimizer nlminb. [5]

Parameter Transformations

Three parameter transformations were performed during this analysis to facilitate modeling: scalar transform of the positivity rate, linear scaling of the prevalence, and log transformation of the county population.

Although the COVID-19 positivity rate varies between (and includes) 0 to 1, beta regression does not allow modelling of proportions that include 0 or 1. Thus it is necessary to use a scalar transform of the positivity rate to map the positivity rate to the range between 0 and 1, but exclusive of both 0 and 1. A commonly used scalar transform is shown in Equation 7, in which y_{init} and y_{transf} are the original and transformed COVID-19 positivity rates respectively, and n is the size of the COVID-19 positivity rate dataset. [6] It is assumed that this scalar transform does not significantly change the shape of the COVID-19 positivity rate distribution.

$$y_{transf} = \frac{y_{init} * (n-1) + 0.5}{n} \tag{7}$$

Prevalence values are very small (minimum $5.7e^{-5}$), but population values are extremely large (maximum $1.0e^{7}$). This difference of multiple orders of magnitude leads to difficulty with optimizer convergence. The distribution of prevalence appeared to be rightward skewed but without excessive outliers, so prevalence was linearly scaled. The pre and post transformation histograms for prevalence are shown in Figure 1. However, the distribution of population appeared to be rightward skewed and to have a significant outlier by multiple orders of magnitude. Therefore, population was transformed with a logarithmic transform. The pre and post transformation histograms for population are shown in Figure 2. It was assumed that given the nonlinear nature of the beta distribution, these transformations would not significantly alter prevalence or population's relationship with positivity rate, respectively. Of note, prevalence histograms are plotted with 100 bins, and population histograms are plotted with 30 bins.

Final Model

The functional form of the mixed effects beta regression model is shown using R syntax in Equation 8.

$$pos \sim TMAX + (1 \mid cty/DOW) + logpop + prevscaled \quad (8)$$

The variable pos represents the COVID positivity rate, TMAX represents the maximum ambient temperature, cty represents the county, DOW represents the day of the week, logpop represents the logarithmic transform of the population, and prevscaled represents the linearly scaled prevalence. Since every day of the week and every county was considered when performing the analysis, both day of the week and county were modeled as random categorical effects. It was assumed that any effects of the day of the week (such as different numbers of open testing centers on the weekend relative to weekdays) would vary by county, so day of the week was modeled as random effects nested within county random effects. Other variables such as maximum ambient temperature, logarithmically transformed population, and linearly scaled prevalence were modeled as fixed effects.

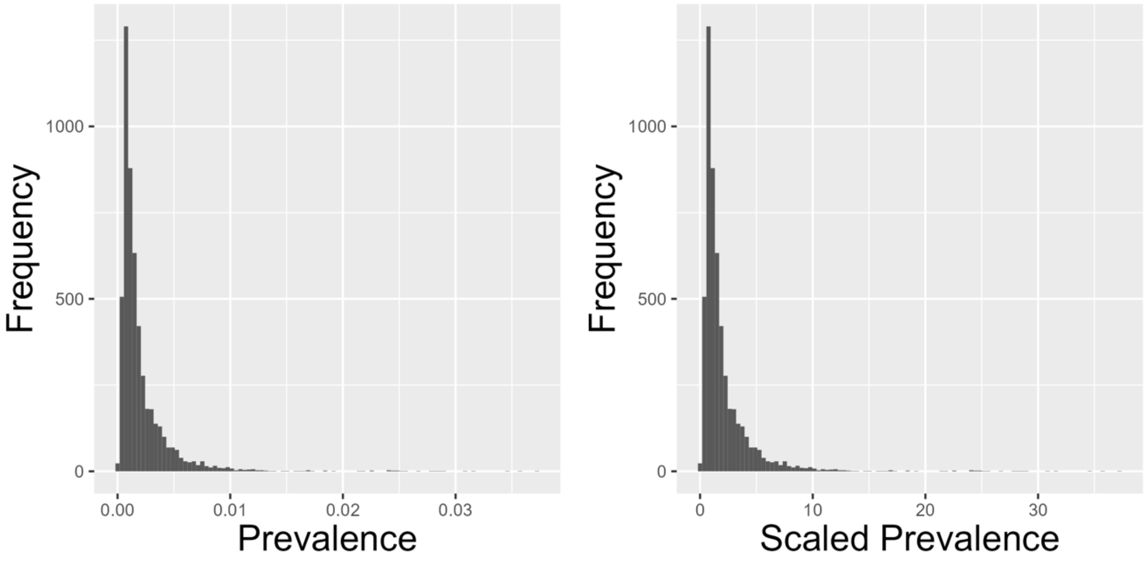

Figure 1: Histograms of raw and scaled prevalence values.

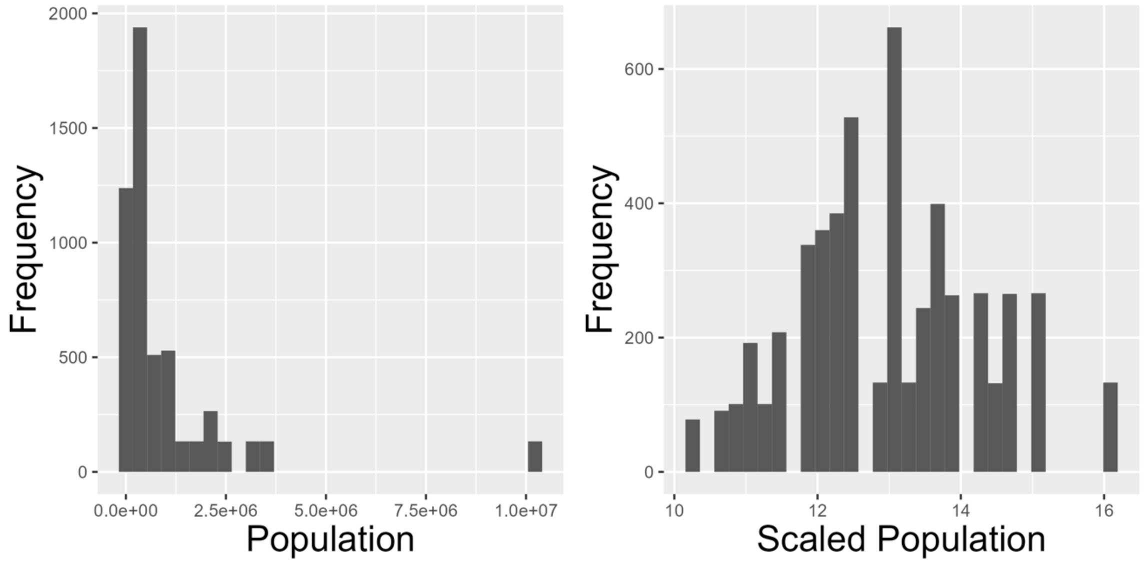

Figure 2: Histograms of raw and scaled population values.

Model Diagnostics

Variation Inflation Factors (VIF)

Briefly, to assess multicollinearity between study variables in regression models, variation inflation factors were calculated. One variation inflation factor (VIF) was calculated for each independent variable using the check_collinearity function from the performance package in R (version 0.10.8) [7]. A VIF value of 1 indicates no multicollinearity of that variable, and low correlation with other predictors occurs at a VIF between 1 and 5. Higher VIF values lead to greater correlation. VIF values are shown in Table 1.

Table 1: Variance Inflation Factors.

	VIF [95% CI]
Maximum Ambient Temperature	1.36 [1.31, 1.41]
Logarithmically Transformed Population	1.00 [1.00, 61.26]
Linearly Scaled Prevalence	1.36 [1.32, 1.41]

When calculated, all VIF values were found to be less than 2, suggesting minimal multicollinearity.

Q-Q Plot

A Q-Q plot of observed vs expected residuals was generated using the DHARMa package in R, and is shown in Figure 3.

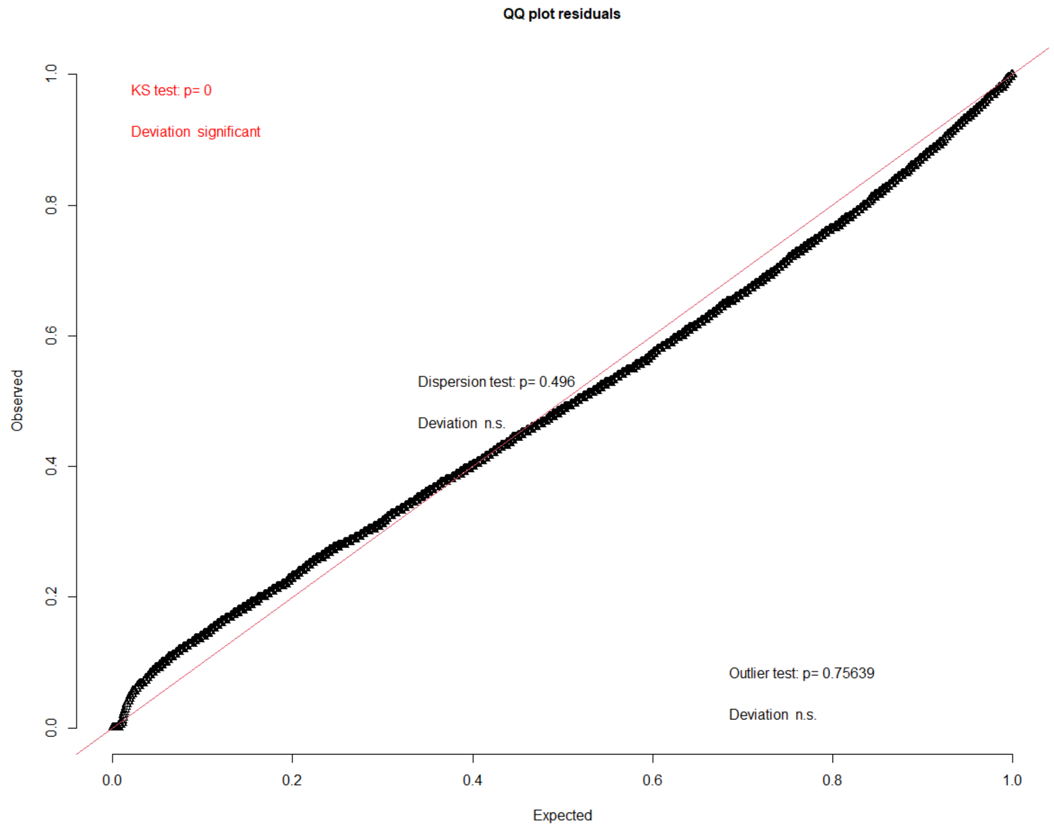

Figure 3: Q-Q plot of observed vs expected residuals.

A one-sample Kolmogorov-Smirnov test was conducted comparing the observed and expected distributions using a two-sided hypothesis, resulting in a D-value of 0.05 and a p-value <0.001. However, the large size of the dataset is expected to drive statistical significance of normality tests, even in cases of small differences in expected distribution. The overall minimal deviation of the residuals from normality when plotted, and non-significant results of the outlier and dispersion tests (below) suggest the underlying residual distribution is sufficiently close to normal for the validity of this analysis.

Outlier Test

A histogram of residuals was plotted to assess for outliers, as shown in Figure 4.

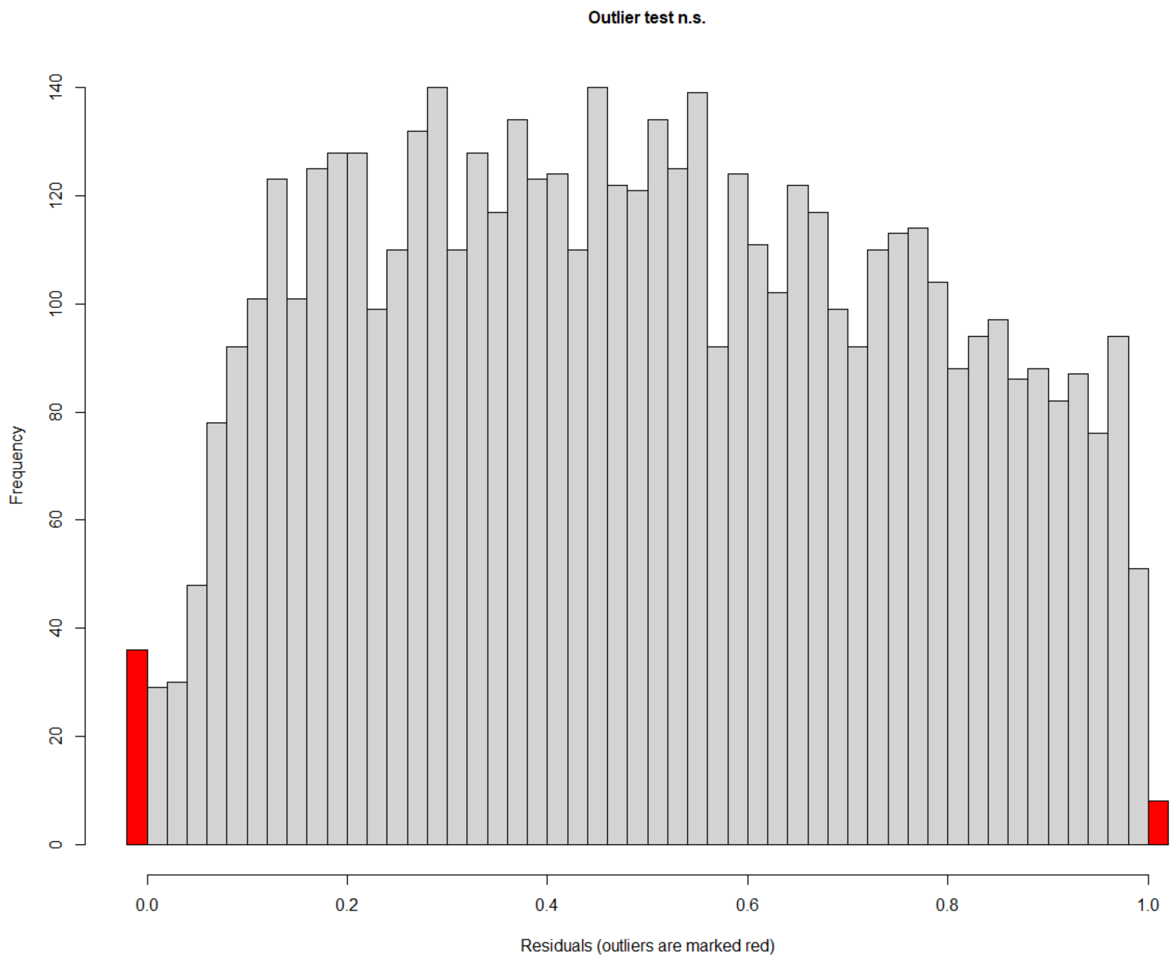

Figure 4: histogram of residuals to assess for outliers.

Visually there are minimal outliers. The binomial outlier test for continuous distributions was conducted. The frequency of outliers was found to be 0.0083, which was not significantly different from the expected frequency of 0.0080 (95% confidence interval 0.0061 to 0.0112), and the p-value was found to be 0.76. Overall this suggests there are neither too few nor too many outliers present.

Dispersion Plot

A dispersion plot of the standard deviations of the simulated residuals (the histogram) with the standard deviation of the fitted residuals indicated as a red line is shown in Figure 5.

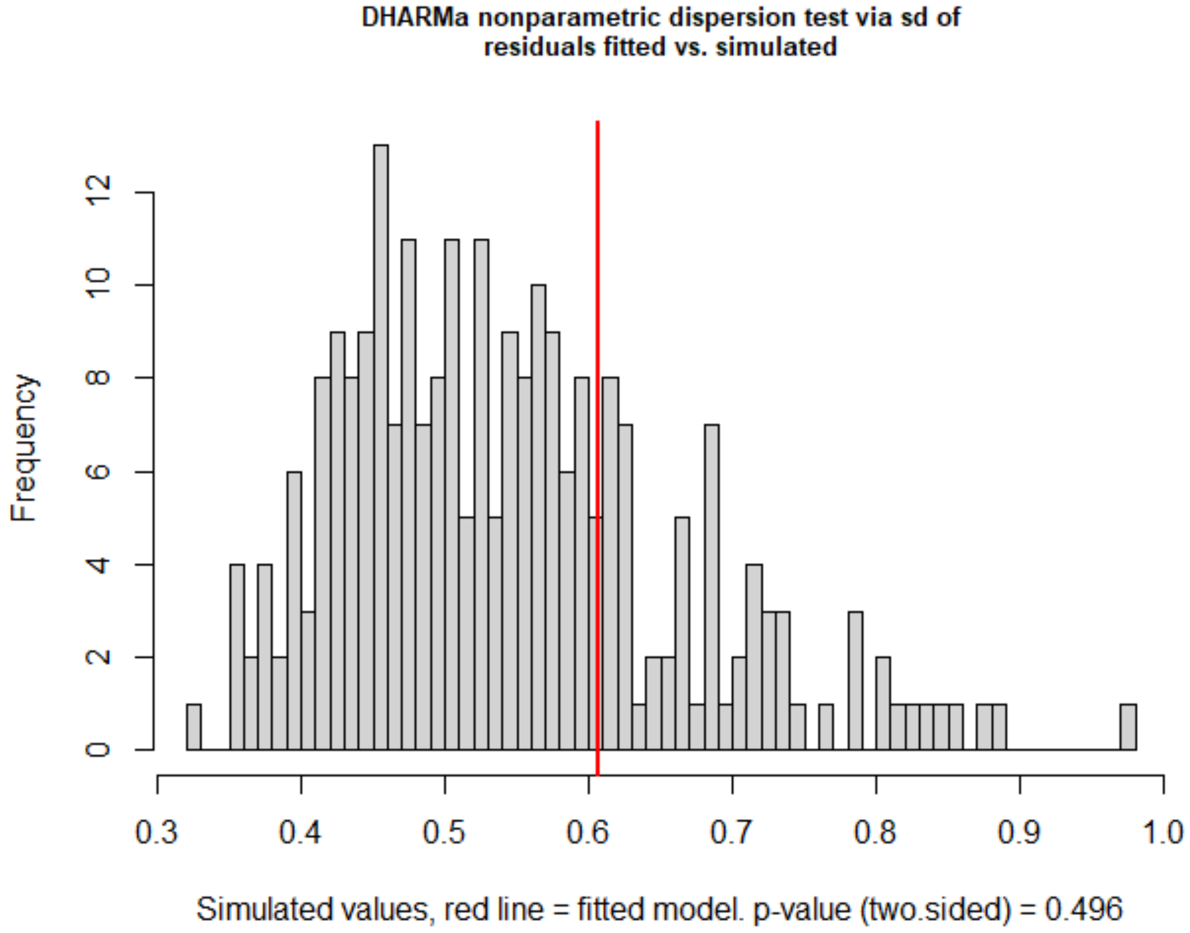

Figure 5: Nonparametric dispersion plot showing a histogram of standard deviations for simulated residuals, and a red line indicating the standard deviation for the fitted residuals.

A nonparametric dispersion test using the standard deviation of fitted and simulated residuals was performed using a two-sided alternative hypothesis. The dispersion was found to be 1.12 with a p-value of 0.5, indicating that the data is neither overdispersed nor underdispersed.

Residual vs Predictor Plots

Fitted Pearson residuals were calculated, new data generated, and simulated residuals calculated using the DHARMa package in R (version 0.4.6). 250 simulations were used to simulate the new data, and all hierarchical levels within the model were simulated. Simulated residuals were then plotted against predictor variables (maximum ambient temperature, scaled prevalence, and logarithmically transformed population) in figures 6-8.

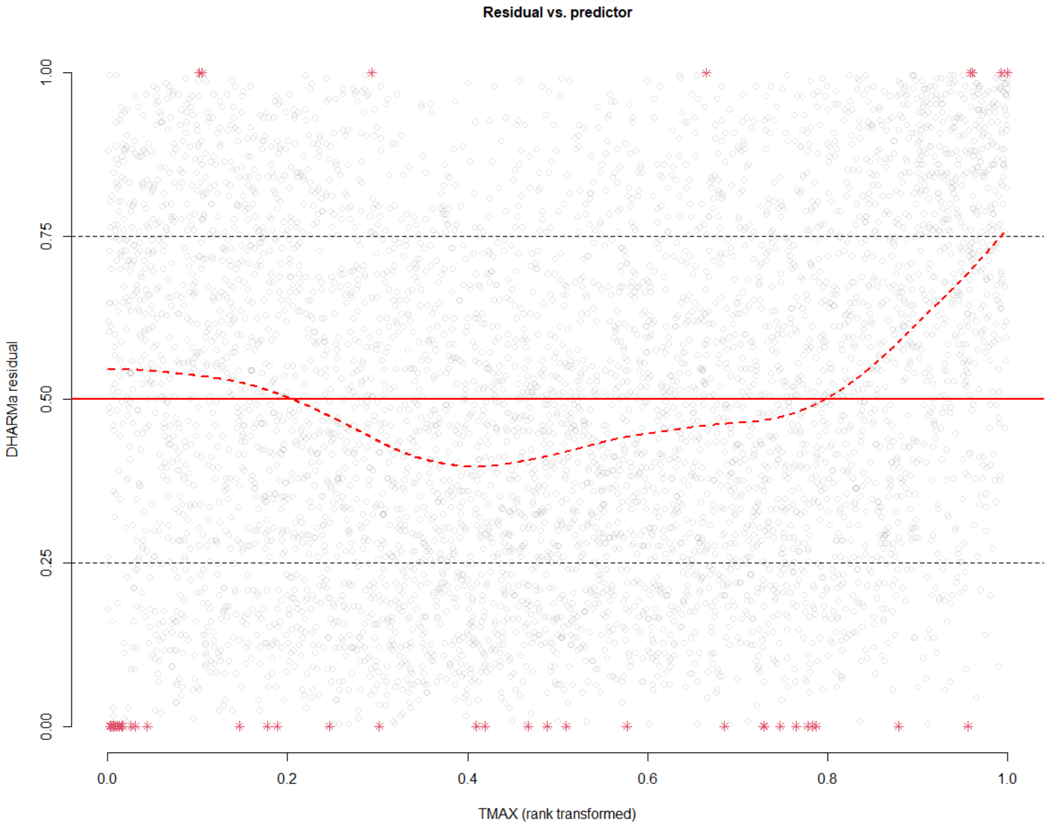

Figure 6: plot of simulated residuals vs maximum ambient temperature (°F).

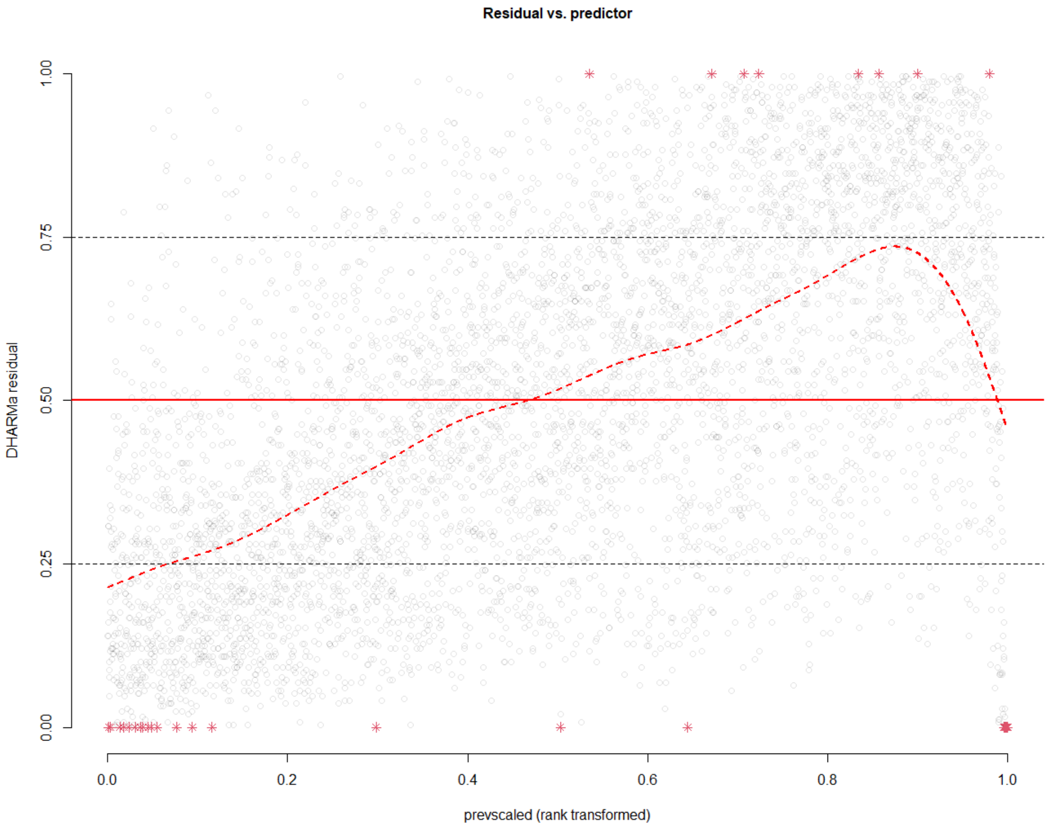

Figure 7: plot of simulated residuals vs scaled prevalence.

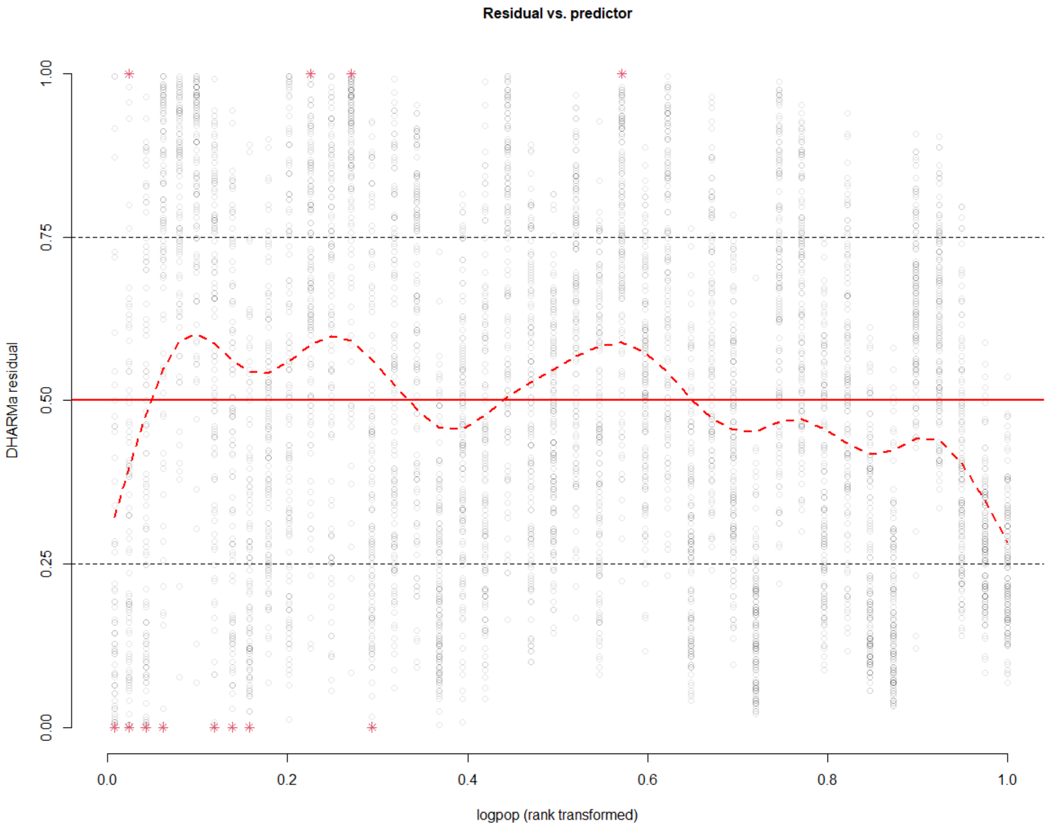

Figure 8: plot of simulated residuals vs logarithmically transformed population.

Fitted residuals notably were less than 3 overall, and only 3 data points had residuals greater than 1.5. The residual vs predictor plots were notable for minimal outliers, no clustering of outliers, significant spread of residuals, and minimal trend in the residuals with change in the predictors. The residuals appear to increase mildly with increasing prevalence, but this trend is overshadowed by the large spread of the residuals overall. Overall, the residuals suggest that the beta regression model has reasonable model fit.

Overall, these tests (VIF, residual vs predictor plots, K.S. test, outlier test, and dispersion test) support the robustness and stability of the beta regression model in modeling COVID-19 positivity rate.

References

1. Rdocumentation. cor.test: Test for Association/Correlation Between Paired Samples. Available from: https://www.rdocumentation.org/packages/stats/versions/3.6.2/topics/cor.test [accessed Sept 24, 2024].
2. Dohm, M. 16.4: Spearman and other correlations. LibreTexts Statistics. Available from: https://stats.libretexts.org/Bookshelves/Applied_Statistics/Mikes_Biostatistics_Book_(Dohm)/16%3A_Correlation%2C_Similarity%2C_and_Distance/16.4%3A_Spearman_and_other_correlations [accessed Sept 24, 2024].
3. Dohm, M. 16.1: Product-moment correlation. LibreTexts Statistics. Available from: https://stats.libretexts.org/Bookshelves/Applied_Statistics/Mikes_Biostatistics_Book_(Dohm)/16%3A_Correlation%2C_Similarity%2C_and_Distance/16.1%3A_Product-moment_correlation [accessed Sept 24, 2024].
4. Cribari-Neto, F. and Zeileis, A. Beta Regression in R. Available from: https://cran.r-project.org/web/packages/betareg/vignettes/betareg.html [accessed Sept 24, 2024].
5. Rdocumentation. glmmTMB (version 1.1.5). Available from: https://www.rdocumentation.org/packages/glmmTMB/versions/1.1.5 [accessed Sept 24, 2024].
6. Smithson M, Verkuilen J. A Better Lemon Squeezer? Maximum-Likelihood Regression with Beta-Distributed Dependent Variables. Psychological Methods. 2006; 11(1), 54–71. doi: 10.1037/1082-989X.11.1.54
7. Rdocumentation. check_collinearity: Check for multicollinearity of model terms. Available from: https://www.rdocumentation.org/packages/performance/versions/0.12.1/topics/check_collinearity [accessed Sept 24, 2024].
